# Perinatal outcomes of intrauterine fetal arrhythmias: A 10-year retrospective cohort study

**DOI:** 10.1097/MD.0000000000033244

**Published:** 2023-03-10

**Authors:** Qing Hu, Hua Liao, Tingting Xu, Hongyan Liu, Xiaodong Wang, Haiyan Yu

**Affiliations:** a Department of Obstetrics and Gynecology, West China Second University Hospital, Sichuan University, Chengdu, China; b Key Laboratory of Birth Defects and Related Diseases of Women and Children (Sichuan University), Ministry of Education, Chengdu, China.

**Keywords:** auto-immune diseases, fetal arrhythmias, fetal cardiac structural malformations, hydrops fetalis, intrauterine therapy

## Abstract

Sustained fetal arrhythmia can produce life-threatening fetal distress, fetal hemodynamic compromise, hydrops fetalis, or even fetal death. Survivors may subsequently possess severe neurologic deficits. We conducted a retrospective observational study of pregnant women hospitalized with fetal arrhythmias from January 2011 to May 2020 at West China Second University Hospital, and fetal arrhythmias were diagnosed by specialists in cardiac ultrasonography. Of 90 cases of fetal arrhythmias, 14 (15.6%) were complicated by fetal congenital heart disease (CHD), 21 (23.33%) by fetal-hydrops, 15 (16.67%) cases by intrauterine therapy, and 6 (6.67%) by maternal auto-immune disease. In the fetal-hydrops group, the intrauterine therapy rate was significantly higher (47.62% vs 7.24%, *P* < .001) and the survival rate significantly lower (47.62% vs 92.75%, *P* < .001) than in the nonfetal hydrops group. A fetus whose arrhythmia was complicated by fetal-hydrops and CHD was delivered earlier and exhibited a lower cardiovascular profile score at diagnosis and birth, lower birth weight, and a higher rate of pregnancy termination than cases without hydrops and CHD (*P* < .05). Among the cases with maternal auto-immune disease, 71.43% (5/7) manifested fetal atrioventricular block. Multiple linear regression analysis revealed that 3 variables – fetal-hydrops (*P* < .001), body mass index (*P* = .014), and gestational age at diagnosis of fetal arrhythmia (*P* = .047) – were correlated with the gestational delivery age of arrhythmic fetuses. Parents should be counseled by the multidisciplinary team regarding the individualized management and prognosis of the arrhythmic fetus, and individualized fetal intrauterine therapy should be performed if necessary.

## 1. Introduction

The normal fetal heart rate is within the range of 110 to 160 bpm, and is considered abnormal if it is either outside this normal range or if the rhythm is irregular, and it can be classified into 3 categories: irregular arrhythmia, tachyarrhythmia, or bradycardia.^[1,2]^ Fetal arrhythmia can also be classified as sustained or intermittent according to whether the arrhythmia was present for more than 50%^[3,4]^ of the time during echocardiographic examination.

The clinical incidence of fetal arrhythmia is between 1% and 2%,^[3,5]^ but the incidence in pregnant women at high risk is approximately 16.6% between 21 gestational weeks and full term. Sinus tachycardias and the most common fetal arrhythmia, premature atrial contraction (PAC), are both benign arrhythmias.^[6]^ Despite the low clinical significance of PAC, approximately 1% of PAC cases are complicated by structural heart disease, and approximately 0.5% progress to supraventricular tachycardia (SVT). Sustained fetal arrhythmia can lead to fetal hemodynamic abnormalities that frequently result in fetal cardiac dysfunction, fetal-hydrops, fetal distress, or even fetal death. Arrhythmias that continue until the postnatal period and that demonstrate hemodynamic changes require intervention,^[6]^ and it has been reported that 10% to 20% of cases referred to fetal cardiology specialists possess fetal arrhythmias.^[7]^

During pregnancy, fetal arrhythmias are diagnosed by fetal echocardiography when abnormal auscultation of the fetal heart is detected at antenatal visits or during ultrasonographic examination.^[1,8]^ The rate, duration, and origin of the rhythm and the degree of irregularity are often closely associated with the potential for hemodynamic consequences.^[1]^ For example, the earlier the onset of tachycardia and the faster the ventricular rhythm, the more likely it is that fetal-hydrops will develop.^[1]^ Surviving patients may also suffer severe neurologic deficits.^[5,6]^ A ventricular rate < 55 bpm is now recognized to constitute a significant predictive risk factor for high mortality rate.^[6]^

The trans-placental passage of maternal antiRo/antiLa antibodies generates passively acquired autoimmunity that may be associated with serious fetal complications, including inflammation, fibrosis, fetal auto-immune complete heart block, and calcification of the conduction system.^[9,10]^

Although several authors have previously investigated fetal arrhythmias, few have reported on the outcomes of fetal arrhythmia during pregnancy or their long-term follow-up after birth. Hence, as preterm birth is the principal cause of neonatal death,^[11]^ we herein performed a 10-year retrospective cohort study on pregnant women with fetal arrhythmias to establish which factors were associated with gestational age at delivery of babies with a prenatal diagnosis of fetal arrhythmia. We present the following article in accordance with the STROBE guidelines, propose a management strategy for this condition.

## 2. Methods

### 2.1. Study design and participants

**Figure 1.**
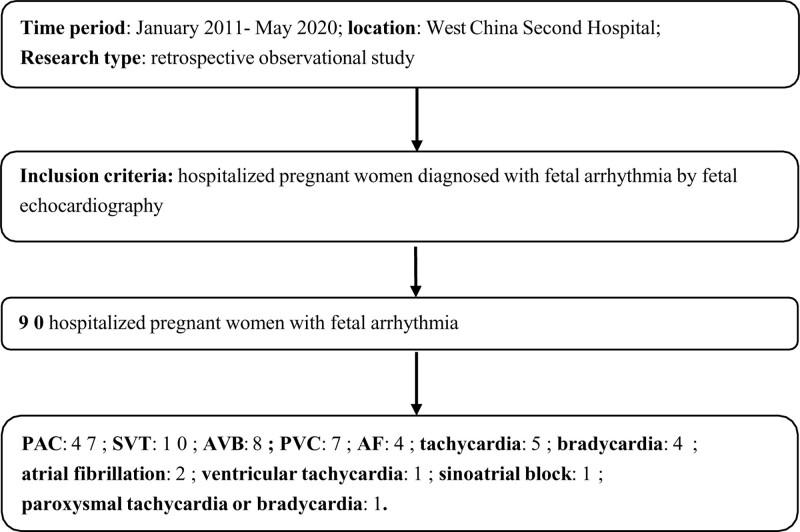
Flowchart of the study.

We conducted an observational study of the retrospective cohort type of fetal arrythmia as presented in pregnant women who were hospitalized at the West China Second University Hospital between January 2011 and May 2020. This was not a screening study. Some patients underwent prenatal care at our hospital and some were referred from secondary local centers due to fetal arrhythmia. The inclusion criteria included hospitalized pregnant women whose fetus was diagnosed with arrhythmia via fetal echocardiography. The flow chart of this study is shown in Figure 1.

### 2.2. Fetal assessments

Fetal echocardiography was performed by cardiac ultrasound specialists when abnormal auscultation of the fetal heart was detected at antenatal visits or during ultrasonographic examination.^[8,12]^ Fetal cardiac function was also assessed using the fetal cardiovascular profile score that encompassed fetal-hydrops, abnormal venous and arterial Doppler findings, cardiomegaly, atrioventricular valve regurgitation, and cardiac dysfunction; each component was scored 1 to 2 points according to ultrasonographic examination.^[13]^

Fetal-hydrops was defined as the accumulation of abnormal fluid in at least 2 different fetal compartments, and included pleural effusion, pericardial effusion, and peritoneal effusion; skin edema (skin thickness > 5 mm); thickened placenta (≥4 cm in the second trimester or ≥ 6 cm in the third trimester); or polyhydramnios.^[14,15]^ Fetal-hydrops was monitored every other 3-to-5-day period during pregnancy according to the intrauterine state of the arrhythmic fetus.^[16]^

### 2.3. Maternal assessments

Following a diagnosis of fetal arrhythmia, TORCH (toxoplasmosis, other [syphilis], rubella, cytomegalovirus, herpes simplex virus) and thyroid function were evaluated in maternal peripheral blood. Pregnant women with fetal arrhythmia were hospitalized so that they could be more closely observed for signs of fetal compromise or distress. Early delivery was considered and weighed against the complications of prematurity and fetal status in utero.

### 2.4. Intrauterine therapy

In cases requiring intrauterine treatment, pregnant women were evaluated by cardiac ultrasonography, electrocardiogram, cortisol assay, and levels of blood electrolytes before the initiation of drug treatment. The choice of trans-placental drugs was made by referring to the statement of the American Heart Association^[16]^: digoxin and sotalol were used in most tachycardia cases, while observation and internal treatment of maternal auto-immune diseases were implemented in irregular and bradycardia cases. During the administration of trans-placental drugs, fetal and maternal conditions were closely monitored. In addition, fetal echocardiography was performed every 5 to 7 days.

### 2.5. Data collection

We initially compiled data regarding fetal arrhythmia diagnosis, treatment, and maternal-neonatal outcomes. The collected data encompassed maternal complications, history of drug use during pregnancy, gestational age at fetal arrhythmia diagnosis, fetal intrauterine complications, fetal congenital heart disease (CHD), gestational age at delivery, admission to the NICU, neonatal conditions, and short- and long-term complications after birth.

### 2.6. Statistical analyses

The variables we examined included maternal age, body mass index (BMI), gravity, parity, intrahepatic cholestasis of pregnancy, gestational diabetes mellitus, hypothyroidism, gestational age when fetal arrhythmias were diagnosed, gestational age at birth, fetal-hydrops, fetal CHD, and birth weight. The primary outcome was survival of the arrhythmic fetus. Variables were analyzed by exploiting multiple linear regression; and chi-squared, Mann–Whitney *U*, and Kruskal–Wallis *H* tests were applied where appropriate. *P* values < .05 were considered to be statistically significant. All statistical analyses were executed using SPSS (version 23.0, IBM, 2015), and Figures were generated using GraphPad Prism (Version 9.0.0[121], GraphPad Software, LLC).

## 3. Results

We admitted 90 pregnant women with arrhythmic fetuses to our hospital between January of 2011 and May of 2020, detailed clinical reports were generated. The characteristic arrhythmias, associated fetal and maternal medical conditions, and pregnancy outcomes are described below.

### 3.1. Fetal arrhythmias in admitted pregnant women

Of the 90 cases of fetal arrhythmia, 5 had twin pregnancies: 3 dichorionic diamniotic (DCDA) twins, 1 case of monochorionic monoamniotic (MCMA) twins, and 1 case of monochorionic diamniotic (MCDA) twins (the demographic and obstetric characteristics of the admitted women are depicted in Table 1). Of the admitted patients, 14 (15.6%) cases were complicated by fetal CHD (these are displayed in Table 2), 21 (23.33%) by fetal-hydrops, 15 (16.67%) by intrauterine therapy, and 6 (6.67%) by maternal auto-immune disease. The types of fetal arrhythmias among the 90 cases are presented in Figure 1. Of the 5 twin pregnancies, 4 cases were complicated by 1 twin with arrhythmia with the co-twin normal (1 twin showed PAC in 1 monochorionic diamniotic twin pregnancy, 1 in dichorionic diamniotic twin (DCDA twin) pregnancy, and 1 in monochorionic monoamniotic twin pregnancy; and 1 twin manifested tachycardia in 1 DCDA twin pregnancy), and both twins were noted to have 1-degree atrioventricular block (AVB) in 1 DCDA twin pregnancy.

**Table 1:** Demographic and obstetric characteristics of women whose pregnancies were complicated by fetal arrythmias and who were assigned to this study (n = 90).

Age (yr)	
Mean ± SD	29.71 ± 5.60
Range	19–44
Body mass index (kg/m^2^)	
Mean ± SD	25.62 ± 2.72
Range	20.8–33.34
Singleton pregnancy (%)	85 (95.44%)
Twin pregnancy	5 (5.56%)
IVF-ET	12 (13.33%)
Spontaneous conception	78 (86.67%)
GA at diagnosis of fetal arrhythmia (w)	
Mean ± SD	30.43 ± 5.78
Range	11^+6^–40^+1^
Maternal complications	
ICP	13 (14.44%)
GDM	17(8.89%)
Hypothyroidism	9 (10.00%)
Maternal auto-immune disease	6 (6.67%)
TOP	12
Neonatal deaths	1
Intrauterine death	2

GDM = gestational diabetes mellitus, ICP = intrahepatic cholestasis in pregnancy, IVF-ET = in vitro fertilization and embryo transfer, SD = standard deviation, TOP = termination of pregnancy.

**Table 2:** Cases of fetal arrhythmias combined with fetal congenital heart disease.

No.	Fetal CHD	Fetal arrhythmias	Arrhythmia diagnosis GA (w)	Fetal intrauterine complication	Intrauterine therapy	Birth w	Birth weight (g)	Outcome
1	ASD	AF	27^+6^	Valvular regurgitation, generalized cardiac enlargement, pericardial effusion	Y	32^+1/^	/	TOP
2	VSD	PAC	37^+1^	None	N	/	/	Requested discharge, refused follow-up
3	VSD	PVC	26	Pericardial effusion	N	27^+2^	/	TOP
4	VSD	PVC	25	None	N	39^+2^	3090	In good health
5	VSD	Third-degree AVB	23^+5^	Valvular regurgitation, pericardial effusion	N	23^+5^	/	TOP
6	VSD	Bradycardia	30	None	N	38^+3^	3070	Died at 2 mo of age
7	AVSD	PAC	35	None	N	39^+4^	4070	In good health
8	Atrial septal aneurysm	PAC	31^+1^	None	N	38^+5^	2550	Failed long-term follow-up
9	Atrial septal aneurysm	PAC	36^+5^	Giant lymph cyst of left thoracic wall	N	40	3070	Failed long-term follow-up
10	Atrial septal aneurysm	SVT	32	None	N	41^+1^	2960	In good health
11	Ductus arteriosus hemangioma	PAC	37^+6^	None	N	40^+3^	3080	In good health
12	Pulmonary artery valve stenosis	Third-degree AVB	30	Pericardial effusion, valvular regurgitation	Y	23^+5^	/	TOP
13	Ectopic drainage of superior vena cava	AF	30	None	Y	39^+4^	4010	In good health
14	Univentricular heart, ectopic great arteries	PAC	31^+1^	None	N	31^+4^	/	TOP

AF = atrial flutter, ASD = atrial septal defect, AVB = atrioventricular block, AVSD = atrial ventricular septal defect, CHB = complete heart block, CHD = congenital heart disease, GA = gestational age, PAC = premature atrial contraction, PVC = premature ventricular contractions, SVT = supraventricular tachycardia, TOP = termination of pregnancy, VSD = ventricular septal defect, Y = yes.

We collected the detailed data and information from the 90 cases. Thirteen cases ended in termination of the pregnancy; 2 cases underwent induced labor due to fetal death; and 75 cases resulted in live births, of which 74 newborns survived.

### 3.2. Perinatal outcomes

#### 3.2.1. Perinatal outcomes of the fetal-hydrops and nonfetal hydrops groups.

Of the 90 cases, 21 cases were included in the fetal-hydrops group and 69 cases in the nonfetal hydrops group, depending on whether hydrops occurred during pregnancy.

#### 3.2.2. Fetal-hydrops group.

In the fetal-hydrops group, fetal arrhythmia was diagnosed at 27.68 ± 3.79 (range, 21–35) weeks of gestation, with a mean fetal cardiovascular profile score (CVPS) of 8.15 ± 1.14 (range, 6–10). Four cases exhibited CHD, and 10 cases were treated with trans-placental drugs (primarily digoxin).

Fetal death occurred in 2 cases, and 9 women chose to terminate their pregnancies. Ten women experienced live births, with a delivery age at 36.26 ± 1.77 (range, 33–38^+4^) weeks of gestation, with a mean fetal CVPS of 8.63 ± 0.92 (range, 8–10) before birth. Mean birth weight was 2541.00 ± 890.53 (range, 1260–3420) g, with no cases demonstrating amniotic fluid turbidity. Four cases were transferred to the NICU. Ten newborns survived (1 case was a DCDA twin pregnancy), the outcomes were as follows. Three cases showed a restored sinus rhythm in utero; 1 case was treated with gamma globulin protein in the NICU and developed well at follow-up; 1 case was prescribed digoxin and sotalol for 6 months and was regularly visited by a pediatric cardiologist (the child is developing well, with no arrhythmic disease); 1 case had a restored sinus rhythm at 10 days of age; 1 case was prescribed propafenone and sotalol to control heart rhythm and was to undergo cardiac radiofrequency ablation at 3 years of age; and 3 cases required no regular pediatric care and developed well, with no arrhythmic disease at follow-up.

#### 3.2.3. Nonfetal hydrops group.

In the nonfetal hydrops group, fetal arrhythmia was diagnosed at 31.27 ± 6.03 (range, 11^+6^–40^+1^) weeks of gestation, with a fetal CVPS of 9.68 ± 0.96 (range, 5–10). PAC was the primary fetal arrhythmia type (62.3%, 43/69). Nine cases had CHD, 5 cases were treated with trans-placental drugs (primarily digoxin), and 4 women terminated their pregnancies.

Sixty-five cases resulted in live births, with a delivery age at 38.65 ± 1.14 (range, 32–42^+2^) weeks of gestation, with a fetal CVPS before birth of 9.85 ± 0.54 (range, 7–10). The mean birth weight was 3192.38 ± 590.28 g (range, 1690–5250 g), with 2 cases demonstrating amniotic fluid turbidity. In 21 cases the neonates were transferred to the NICU. One infant with fetal bradycardia died at 2 months old due to severe pneumonia, ventricular septal defect (VSD), cardiac insufficiency, respiratory failure, sinus bradycardia with arrhythmia, and myocardial injury. Sixty-four newborns survived.

Among the survivors, 18 cases were lost to long-term follow-up, while the remaining 46 cases were followed up by a pediatric cardiologist without issue. The long-term outcomes of the latter cases were as follows: 23 fetuses exhibited a restored sinus rhythm in utero; 1 fetus had a restored sinus rhythm before birth; 2 cases recovered heart rhythm at birth; 1 case was treated with metoprolol after birth and rhythm was restored at 8 months; 1 case is preparing to undergo surgery; 1 case underwent VSD surgery at 6 months, and sinus rhythm was successfully restored as a result; 1 case was lost to follow-up after discharge from the NICU; 1 case underwent cardiac surgery due to patent ductus arteriosus; 10 cases showed a restored sinus rhythm – 3 cases at 1 month, 2 at 3 months, 1 at 5 months, 2 at 6 months, and 2 at 1 year each; and the remaining 5 cases did not require regular pediatric care and have all developed well, with no arrhythmic disease at follow-up.

**Figure 2.**
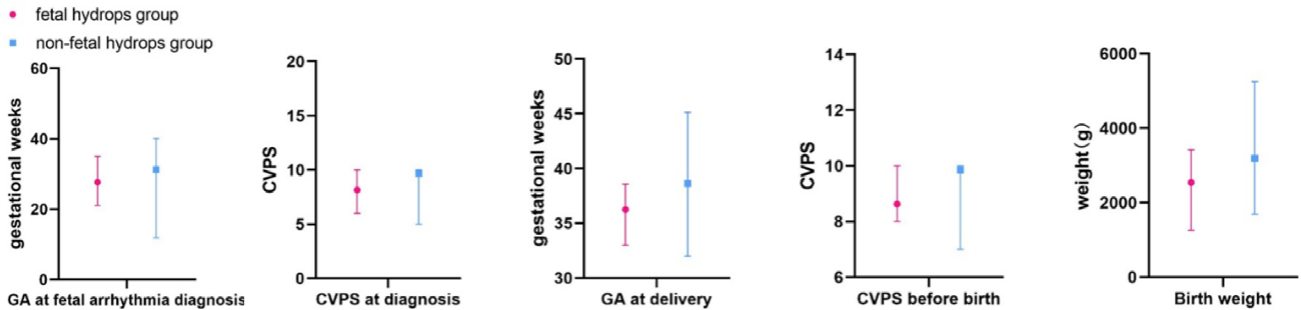
Demographic characteristics of the fetal-hydrops and nonfetal hydrops groups.

The survival rate in the fetal-hydrops group was significantly lower than that in the fetal nonhydrops group (47.62% vs 92.75%, *P* = .000), and the rate of intrauterine therapy in the fetal-hydrops group was significantly higher than that in the fetal nonhydrops group (47.62% vs 7.24%, *P* = .000). The detailed data are shown in Table 3 and Figure 2.

**Table 3:** Demographic characteristics of groups with fetal-hydrops and nonfetal hydrops.

	Fetal-hydrops	Nonfetal hydrops	*P*
Total	21	69	
GA at diagnosis of fetal arrhythmia (w)	27.68 ± 3.79	31.27 ± 6.03	.001
CVPS at diagnosis	8.15 ± 1.14	9.68 ± 0.96	.000
Fetal death, n (%)	2 (9.52)	0 (0)	.000
TOP, n (%)	9 (42.86)	4 (5.8)	.000
CHD, n (%)	4 (19.05)	9 (13.04)	.000
Trans-placental drugs, n (%)	10 (47.62)	5 (7.24)	.000
Live birth, n (%)	10 (47.62)	65 (94.2)	.000
GA at delivery (w)	36.26 ± 1.77	38.65 ± 1.14	.000
CVPS before birth	8.63 ± 0.92	9.85 ± 0.54	.000
Birth weight (g)	2541.0 ± 890.53	3192.38 ± 590.28	.010
NICU, n (%)	4 (40)	21 (32.31)	.003
Survivor, n (%)	10 (47.62)	64 (92.75)	.000
Neonatal death, n	0	1	.000

CHD = congenital heart disease, CVPS = cardiovascular profile score, GA = gestational age, TOP = termination of pregnancy.

#### 3.2.4. Comparison among subgroups complicated with fetal-hydrops or CHD.

Four arrhythmia cases showed combined fetal-hydrops and CHD. Median gestational age at fetal arrhythmia diagnosis was 28.94 weeks (IQR, 27.41–30.04), the median CVPS at diagnosis was 6 (IQR, 4.75–7.5), the median gestational age at delivery was 30.86 (IQR, 29.32–31.82) weeks, the median CVPS before birth was 6 (IQR, 5–7.5), and the median birth weight was 1780 g (IQR, 1541.25–2031.75 g). No fetal deaths occurred and all women terminated their pregnancies, although intrauterine therapy was implemented in 2 cases (50%).

Seventeen cases showed combined fetal-hydrops but no CHD. Median gestational age at fetal arrhythmia diagnosis was 29 weeks (IQR, 25.5–30.79), the median CVPS at diagnosis was 8 (IQR, 7–8.76), the median gestational age at delivery was 36.72 weeks (IQR, 35.05–37.93), the median CVPS before birth was 8.5 (IQR, 6–10), and the median birth weight was 2740 g (IQR, 2060–2930 g). Fetal death occurred at a rate of 11.76%, and 7 women (41.18%) chose termination of pregnancy (TOP). Trans-placental drugs were used in 8 cases (47.06%), 10 cases (58.82%) experienced live births, and 4 infants (40%) were transferred to the NICU. All babies born live survived.

We noted 9 cases with no fetal-hydrops but with CHD. The median gestational age at fetal arrhythmia diagnosis was 30 weeks (IQR, 25–32), the median CVPS at diagnosis was 10 (IQR, 9–10), the median gestational age at delivery was 39.29 weeks (IQR, 38.71–39.57), the median CVPS before birth was 10 (IQR, 10–10), and the median birth weight was 3000 g (IQR, 2550–3090 g). One woman (11.11%) chose TOP and 9 women (100%) produced a live birth. Two infants (22.22%) were transferred to the NICU and 1 (11.11%) was discharged and ultimately died. The rate of survival was 88.89%.

There were 60 cases with no fetal-hydrops or CHD. The median gestational age at fetal arrhythmia diagnosis was 33 weeks (IQR, 27.15–35.22), the median CVPS at diagnosis was 10 (IQR, 9–10), the median gestational age at delivery was 39.14 weeks (IQR, 37.43–39.57), the median CVPS before birth was 9.5 (IQR, 9–10), and the median birth weight was 3240 g (IQR, 2712.50–3477.51 g). Four women (4.35%) chose TOP and 5 (7.25%) underwent intrauterine therapy. Fifty-six babies (81.86%) were born live and 19 cases (27.54%) were transferred to the NICU. All delivered babies survived and no neonatal deaths were noted at follow-up.

**Figure 3.**
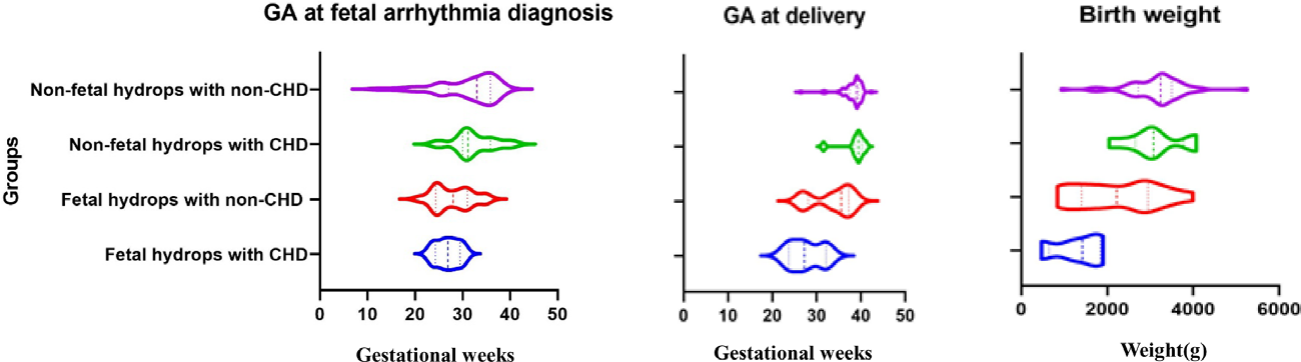
Comparisons of subgroups.

Comparisons were statistically significant and details are shown in Table 4 and Figure 3. The gestational age at which fetal arrhythmia was diagnosed and babies delivered was earliest in the subgroup of fetal-hydrops with CHD (*P* = .032; *P* < .001), CVPS at diagnosis and before birth were the lowest in the subgroup of fetal-hydrops with CHD (*P* < .001 and *P* = .005), and birth weight was lowest in the subgroup of fetal-hydrops with CHD (*P* = .010). The TOP rate was significant as well at *P* < .001.

**Table 4:** Comparisons of subgroups.

	Fetal-hydrops with CHD	Fetal-hydrops with nonCHD	Nonfetal hydrops with CHD	Nonfetal hydrops with nonCHD	*P*
Total, n	4	17	9	60	/
GA at diagnosis of fetal arrhythmia (w)	28.94 (27.41–30.04)	29 (25.5–30.79)	30 (25–32)	33 (27.15–35.22)	.032
CVPS at diagnosis	6 (4.75–7.5)	8 (7–8.75)	10 (9–10)	10 (9–10)	.000
Fetal death, n (%)	0	2 (11.76)	0	0	/
TOP, n (%)	4 (100)	7 (41.18)	1 (11.11)	3 (4.35)	.000
Trans-placental drugs, n (%)	2 (50)	8 (47.06)	0	5 (7.25)	.000
Live birth, n (%)	0	10 (58.82)	9 (100)	56 (81.86)	.000
GA at delivery (w)	30.86 (29.32–31.82)	36.72 (35.05–37.93)	39.29 (38.71–39.57)	39.14 (37.43–39.57)	.000
CVPS before birth	6 (5–7.5)	8.5 (6–10)	10 (10–10)	9.5 (9–10)	.005
Birth weight (g)	1780 (1541.25–2031.75)	2740 (2060–2930)	3000 (2550–3090)	3240 (2712.50–3477.51)	.010
NICU, n (%)	/	4 (40)	2 (22.22)	19 (27.54)	.001
Survivor, n (%)	/	10 (100)	8 (88.89)	56 (81.86)	.000
Neonatal deaths, n (%)	/	0	1 (11.11)	0	/

CHD = congenital heart disease, CVPS = cardiovascular profile score, GA = gestational age, TOP = termination of pregnancy.

#### 3.2.5. Maternal auto-immune disease, fetal arrhythmias, and perinatal outcomes.

Six of the 90 cases involved maternal auto-immune disease combined with fetal arrhythmia, 2 cases with connective tissue disease were treated with prednisone and hydroxychloroquine, 1 case with undifferentiated connective tissue disease did not receive medication, 1 case with systemic lupus erythematosus was treated with prednisone and hydroxychloroquine, and 2 cases had Sjögren syndrome (of whom only 1 case was treated with hydroxychloroquine and methylprednisolone). The types of fetal arrhythmias were as follows: 2 cases of third-degree AVB, 1 case of bradycardia, 1 case of sinoatrial block (SAB), 1 case of second-degree AVB, and 1 case of first-degree AVB in both twins of a DCDA pregnancy (the detailed information is available in Table 5).

**Table 5:** Maternal auto-immune disease and fetal arrhythmias.

No.	Maternal auto-immune disease	Maternal autoantibodies	Fetal arrhythmia	GA at arrhythmia diagnosis (wks)	Fetal intrauterine complication	Fetal intrauterine therapy	W at birth	Birth weight (g)	Outcome
1	CTD	ANA	Third-degree AVB	21	Generalized cardiac enlargement	N	28^+6^	878	Induction due to fetal death
2	Undifferentiated CTD	ANA	PAC	25	None	N	26^+2^	/	TOP
3	CTD	ANA	Both twin first-degree AVB	25	Both twin pericardial effusion	Y	35^+4^	2220/1980	Both twin in good health
4	Sjögren syndrome	SSA, SSB	SAB	24	Pericardial effusion	Y	36^+1^	1860	In good health
5	SLE	ANA, SSA, SSB	Third-degree AVB	25 + 2	Generalized cardiac enlargement; pericardial effusion	N	25^+2^	/	TOP
6	Sjögren syndrome	ANA, SSA, SSB	Second-degree AVB	24^+1^	Pericardial effusion	N	26^+1^	/	TOP

ANA = antinuclear antibody, AVB = atrioventricular block, CTD = connective tissue disease, GA = gestational age, N = no, PAC = premature atrial contraction, SAB = sinoatrial block, SLE = systemic lupus erythematosus, SSA = antiRo antibody, SSB = anti-LA antibody, TOP = termination of pregnancy, w = weeks, Y = yes.

In addition, 4 cases had no definitive diagnosis of auto-immune disease: 1 case of atrial flutter (AF) with positive nuclear antibodies, 2 cases of third-degree AVB with positive nuclear antibodies, and 1 case of PAC with positive antiSSB antibodies.

Complications associated with prematurity may result in neonatal morbidity and mortality, and thus the factors influencing gestational delivery age of arrhythmic fetuses constitutes an important issue. We conducted multiple linear regression to analyze the correlation between gestational age at delivery of arrhythmic fetuses and variables that included maternal age, BMI, gravidity, parity, intrahepatic cholestasis of pregnancy, gestational diabetes mellitus, hypothyroidism, maternal auto-immune disease, gestational age at fetal arrhythmia diagnosis, fetal-hydrops, and fetal CHD. The Durbin-Watson test value generated was 1.802, which verified that the research observations were independent of each other and that the regression model was statistically significant (F = 6.522, *P* < .001, adjusted R^2^ = 0.411). The variables that correlated with gestational age at delivery of arrhythmic fetuses were fetal-hydrops (*P* < .001), BMI (*P* = .014), and gestational age at diagnosis of fetal arrhythmia (*P* = .047).

## 4. Discussion

In the present study, fetal PAC accounted for 52.22% (47/90) of the 90 fetal arrhythmia cases who were admitted to our hospital, confirming the findings of a previous study.^[6]^ Of the 90 hospitalized patients, 15.56% (14/90) possessed fetal structural cardiac malformations as follows: 35.71% with VSD; 21.42% with atrial septal aneurysm; and the remainder with atrial septal defect (ASD), ductus arteriosus malformation, pulmonary artery valve stenosis, ectopic drainage of the superior vena cava, fetal univentricular heart and anomalous artery, and ductus arteriosus hemangioma. Consistent with previous studies,^[3]^ we observed 1 case of fetal PAC with paroxysmal SVT, and 1 case of frequent fetal PAC that progressed to SVT and advanced to fetal tachycardia during intrauterine digoxin treatment.^[3,7,8]^ Although the clinical outcome for PAC is better than that for other fetal arrhythmias, patients with PAC still require close observation to avoid adverse perinatal outcomes.

Fetal tachyarrhythmia refers to a fetal heartbeat > 180 bpm, has an incidence of 0.4% to 0.6%, and is most frequently observed in late pregnancy.^[12,17]^ SVT and AF are the most common fetal tachyarrhythmias, while other types such as fetal sinus tachycardia, atrial tachycardia, and junctional tachycardia can be caused by maternal fever or infection.^[8]^ In our study, 22 cases of fetal tachyarrhythmia were diagnosed, but none was caused by maternal fever or infection. SVT was the most common tachyarrhythmia, accounting for 45.45% (10/22); and this was followed by tachycardia at 22.73% (5/22), AF at 18.18% (4/22), atrial fibrillation at 9.09% (2/22), and ventricular tachycardia at 4.55% (1/22).

Fetal bradycardia can be secondary to intrauterine fetal distress, and is characterized by abnormal function of the sinoatrial node caused by auto-immune antibodies, an abnormal cardiac structure, and long Q-T syndrome.^[1]^ In our study, 49 cases of fetal bradycardia were diagnosed in the outpatient department; of these, 32 presented with fetal AVB, 11 with fetal bradycardia, 5 with sinus bradycardia, and 1 with sinoatrial block.

Maternally administered trans-placental antiarrhythmics should be considered for improved perinatal outcomes. Carberry et al^[18]^ reported that rhythm control was achieved in 76% of SVT cases. In a multicenter trial study, fetal tachyarrhythmia resolved in 89.8% of cases overall and in 75.0% of cases of fetal-hydrops after administration of protocol-defined trans-placental digoxin, sotalol, or flecainide.^[19]^

### 4.1. Fetal-hydrops and perinatal outcomes of fetal arrhythmias

The presence of fetal-hydrops is an important determinant of fetal outcome.^[8,14–16,20,21]^ Fetal-hydrops is defined as excessive extravascular fluid of the fetus, leading to extensive subcutaneous edema and fetal serous effusion. Fetal arrhythmia is a common cause of nonimmune fetal edema that refers specifically to cases that were not caused by red cell alloimmunization, with persistent SVT and AF as the most common signs.^[14]^ It is estimated that 35% to 60% of fetal SVT cases are complicated by nonimmune fetal edema, which may then lead to nonimmune edema with a fetal mortality as high as 17%.^[6,15]^ In the present study, among inpatients with nonimmune fetal edema, 28.57% (6/21) were complicated with AVB, 23.8% (5/21) with SVT, and 14.3% (3/21) with PAC; and the survival rate in the fetal-hydrops group was significantly lower than that in the fetal nonhydrops groups (47.62% vs 92.75%, *P* = .000). We therefore recommend identifying fetal edema as soon as possible in the perinatal period, and that the parents be counseled so as to arrive at a timely clinical decision.

Nonimmune fetal edema induced by fetal arrhythmia should also be distinguished from immune fetal edema caused by other conditions. The Maternal-Fetal Medical Association Guidelines recommended that a fetus with nonimmune edema caused by fetal arrhythmia be delivered naturally at 34 weeks of gestation – if not at term – and that trans-placental drugs be used in the absence of maternal-fetal contraindications.^[16]^ In our study, the delivery ages of the nonimmune fetal edema cases were 33, 33^+6^, 35^+4^, 36, 36^+1^, 36^+5^, 37^+2^, 37^+2^, 38^+1^, and 38^+4^ weeks.

### 4.2. Fetal CHD and perinatal outcome of fetal arrhythmias

Several researchers have reported the outcomes of fetal arrhythmia combined with CHD. Wu et al reported 2 cases of fetal VSD with long Q-T syndrome, ventricular tachycardia, and AVB; their cases did not survive.^[22]^ Furthermore, Rice et al noted that fetal atrial septal aneurysm was highly associated with fetal atrial arrhythmias (*P *< .001),^[23]^ and Morales et al found upon ultrasonographic examination that PAC was readily engendered by an atrial septal aneurysm that squeezed the anterior wall of the atrium.^[24]^ Moreover, some authors reported fetal ASD with complete atrioventricular canal defect, ectopic drainage of the pulmonary vein or vena cava, other cardiac structural malformations such as second-degree AV,^[25]^ and fetal ASD with fetal bradycardia.^[26]^

In the present study, we demonstrated similar findings in 14 cases of fetal heart malformations, of whom 42.86% (6/14) manifested fetal VSD complicated by premature ventricular contractions, atrial PAC with left atrial isomerism, third-degree AVB, and bradycardia. Three cases of fetal arrhythmia were complicated by fetal atrial septal aneurysm, 1 of whom showed PAC; and 2 cases of ASD were also complicated by PAC and AF.

Giorgione et al^[27]^ ascertained that fetuses conceived via in vitro fertilization and embryo transfer were at a significantly greater risk of fetal CHD. In our study, 11.76% (12/102) of the admitted women were pregnant by in vitro fertilization and embryo transfer and 1 case was complicated by fetal atrial septal aneurysm, consistent with the findings of the Giorgione group (8.3% vs 15.56%, *P* = .000).

We observed 7 cases of fetal arrhythmias with positive maternal autoantibodies, with 2 cases (2/6, 33.33%) being third-degree AVB – a higher prevalence than that reported previously.^[28]^ The fetal arrhythmias in our study were detected at 21 to 25^+2^ weeks of gestation. Complete heart block occurring earlier than after 16 weeks in antiRo-La patients is rare, and this finding was congruent with previously published reports.^[9,10]^ Auto-immune diseases such as maternal lupus correlate with complete fetal AVB. Although the incidence of positive maternal autoantibodies with fetal AVB and the effects on perinatal outcome require further study, the diagnosis of fetal AVB should be followed by close monitoring of maternal autoantibodies in order to determine the corresponding maternal and fetal treatments needed to improve fetal outcome.^[1]^

It is essential to improve the survival rate of fetuses with arrhythmia and reduce the associated complications. In this study, multiple linear regression analysis revealed that fetal-hydrops, BMI, and the gestational age at fetal arrhythmia diagnosis were statistically significant independent variables of the gestational age at delivery.

### 4.3. Strength and limitations

For this study we collected 90 cases of fetal arrhythmia from a single center over a 10-year period, and compared fetal arrhythmia cases in fetal CHD and nonfetal CHD groups and between fetal-hydrops and nonfetal hydrops groups. The long-term outcomes of the arrhythmic fetuses after birth were followed, fetal arrhythmia and its associated maternal and fetal factors were analyzed. Our results suggest that fetal-hydrops, and body mass index, and gestational age at diagnosis of fetal arrhythmia were correlated with the gestational delivery age of arrhythmic fetuses.

The limitations to this study lay primarily in its retrospective nature and the fact that some women chose to terminate their pregnancies or declined follow-up regarding the long-term outcomes of the infants.

## 5. Conclusions

Based upon previous data and the findings of the current study, the outcomes of arrhythmic fetuses were affected by fetal CHD, fetal-hydrops, and maternal complications (particularly auto-immune diseases). Once fetal arrhythmias are diagnosed, we recommend that parents be counseled by the multidisciplinary team as to the individualized management and prognosis regarding the fetus. If necessary, individualized fetal intrauterine therapy should commence so as to improve the perinatal and long-term outcomes. Close perinatal management should also be undertaken by clinicians in cases showing both fetal-hydrops and fetal CHD.

Our study findings contribute to our understanding of the decisive risk factors in fetal arrhythmia cases, may assist clinicians in fetal maternal medicine and fetal cardiovascular fields in decision-making in the management of fetal arrhythmias. Further research with a larger number of cases, however, is necessary to determine the relationship between the outcomes and other clinical parameters. We posit that this will facilitate the establishment of more beneficial management regimens for fetal arrhythmia.

## Author contributions

**Conceptualization:** Qing Hu.

**Data curation:** Qing Hu, Hua Liao, Hongyan Liu.

**Formal analysis:** Qing Hu.

**Funding acquisition:** Qing Hu, Haiyan Yu.

**Investigation:** Qing Hu, Tingting Xu, Xiaodong Wang.

**Methodology:** Qing Hu, Hua Liao, Haiyan Yu.

**Project administration:** Qing Hu, Tingting Xu, Xiaodong Wang, Haiyan Yu.

**Resources:** Qing Hu, Haiyan Yu.

**Software:** Qing Hu.

**Supervision:** Xiaodong Wang, Haiyan Yu.

**Validation:** Xiaodong Wang.

**Visualization:** Xiaodong Wang, Haiyan Yu.

**Writing – original draft:** Qing Hu, Haiyan Yu.

**Writing – review & editing:** Haiyan Yu.

## References

[1] Batra AS Balaji S. Fetal arrhythmias: diagnosis and management. Indian Pacing Electrophysiol J. 2019;19:104–9. 30817991 10.1016/j.ipej.2019.02.007 PMC6531664

[2] Yuan SM. Fetal arrhythmias: genetic background and clinical implications. Pediatr Cardiol. 2019;40:247–56. 30478614 10.1007/s00246-018-2008-3

[3] Freire G. Surveillance of fetal arrhythmias in the outpatient setting: current limitations and call for action. Cardiol Young. 2015;25:1590–2. 26675609 10.1017/S1047951115002486

[4] Kumar S Lodge J. Prenatal therapy for fetal cardiac disorders. J Matern Fetal Neonatal Med. 2019;32:3871–81. 29716424 10.1080/14767058.2018.1472224

[5] Moatassim S Touleimat S Hazelzet T. Maternal complications induced by digoxin treatment of fetal tachycardia: a retrospective series of 18 cases. J Gynecol Obstet Hum Reprod. 2018;47:35–8. 29208503 10.1016/j.jogoh.2017.11.013

[6] Ortiz-Garrido A Cuenca-Peiro V Conejo-Munoz L. Fetal arrhythmias: diagnosis, treatment and perinatal outcome. Rev Esp Cardiol (Engl Ed). 2015;68:817–9. 26190547 10.1016/j.rec.2015.05.010

[7] Wacker-Gussmann A Strasburger JF. Diagnosis and treatment of fetal arrhythmia. Am J Perinatol. 2014;31:617–28. 24858320 10.1055/s-0034-1372430 PMC4073210

[8] Carvalho JS. Fetal dysrhythmias. Best Pract Res Clin Obstet Gynaecol. 2019;58:28–41. 30738635 10.1016/j.bpobgyn.2019.01.002

[9] Cuneo BF Ambrose SE Tworetzky W. Detection and successful treatment of emergent anti-SSA-mediated fetal atrioventricular block. Am J Obstet Gynecol. 2016;215:527–8. 27418449 10.1016/j.ajog.2016.07.002

[10] Levesque K Morel N Maltret A. Description of 214 cases of autoimmune congenital heart block: results of the French neonatal lupus syndrome. Autoimmun Rev. 2015;14:1154–60. 26284740 10.1016/j.autrev.2015.08.005

[11] Ream MA Lehwald L. Neurologic consequences of preterm birth. Curr Neurol Neurosci Rep. 2018;18:48. 29907917 10.1007/s11910-018-0862-2

[12] Zanardo V Simbi A Parotto M. Morphine-induced supraventricular tachycardia in near-term fetus. Ital J Pediatr. 2018;44:111. 30249290 10.1186/s13052-018-0570-1 PMC6154430

[13] Statile CJ Cnota JF Gomien S. Estimated cardiac output and cardiovascular profile score in fetuses with high cardiac output lesions. Ultrasound Obstet Gynecol. 2013;41:54–8. 23001941 10.1002/uog.12309

[14] Society for MM Norton ME Chauhan SP. Society for maternal-fetal medicine (SMFM) clinical guideline #7: nonimmune hydrops fetalis. Am J Obstet Gynecol. 2015;212:127–39. 25557883 10.1016/j.ajog.2014.12.018

[15] Yuan SM. Cardiac etiologies of hydrops fetalis. Z Geburtshilfe Neonatol. 2017;221:67–72. 28561210 10.1055/s-0042-123825

[16] Donofrio MT Moon-Grady AJ Hornberger LK. Diagnosis and treatment of fetal cardiac disease: a scientific statement from the American Heart Association. Circulation. 2014;129:2183–242. 24763516 10.1161/01.cir.0000437597.44550.5d

[17] Malhame I Gandhi C Tarabulsi G. Maternal monitoring and safety considerations during antiarrhythmic treatment for fetal supraventricular tachycardia. Obstet Med. 2019;12:66–75. 31217810 10.1177/1753495X18808118 PMC6560838

[18] Carberry T Arzu J Coons D. Postnatal outcomes in infants with a history of fetal supraventricular tachycardia. JACC Clin Electrophysiol. 2022;8:1145–51. 36137720 10.1016/j.jacep.2022.06.003

[19] Miyoshi T Maeno Y Hamasaki T. Antenatal therapy for fetal supraventricular tachyarrhythmias: multicenter trial. J Am Coll Cardiol. 2019;74:874–85. 31416531 10.1016/j.jacc.2019.06.024

[20] Swearingen C Colvin ZA Leuthner SR. Nonimmune hydrops fetalis. Clin Perinatol. 2020;47:105–21. 32000919 10.1016/j.clp.2019.10.001

[21] Hinkle KA Peyvandi S Stiver C. Postnatal outcomes of fetal supraventricular tachycardia: a multicenter study. Pediatr Cardiol. 2017;38:1317–23. 28664446 10.1007/s00246-017-1662-1

[22] Wu MH Hsieh FC Wang JK. A variant of long QT syndrome manifested as fetal tachycardia and associated with ventricular septal defect. Heart. 1999;82:386–8. 10455095 10.1136/hrt.82.3.386 PMC1729158

[23] Rice MJ McDonald RW Reller MD. Fetal atrial septal aneurysm - A cause of fetal atrial arrhythmias. J Am Coll Cardiol. 1988;12:1292–7. 3170974 10.1016/0735-1097(88)92614-9

[24] Morales R Bokowski JW Nguyen H. A proposed etiology for atrial tachyarrhythmia in neonates with atrial septal aneurysms. Pediatr Cardiol. 2019;40:230–3. 30426160 10.1007/s00246-018-2017-2

[25] Baschat AA Gembruch U Knopfle G. First-trimester fetal heart block: a marker for cardiac anomaly. Ultrasound Obstet Gynecol. 1999;14:311–4. 10623989 10.1046/j.1469-0705.1999.14050311.x

[26] Xiao Hong XU Xi Pin Z. Children bradycardia caused by atrial septal defect since fetus: a case report. J Qinghai Coll. 2006;27:142.

[27] Giorgione V Parazzini F Fesslova V. Congenital heart defects in IVF/ICSI pregnancy: systematic review and meta-analysis. Ultrasound Obstet Gynecol. 2018;51:33–42. 29164811 10.1002/uog.18932

[28] Wainwright B Bhan R Trad C. Autoimmune-mediated congenital heart block. Best Pract Res Clin Obstet Gynaecol. 2020;64:41–51. 31685414 10.1016/j.bpobgyn.2019.09.001

